# Occurrence of Biogenic Amines in Wines from the Central European Region (Zone B) and Evaluation of Their Safety

**DOI:** 10.3390/foods12091835

**Published:** 2023-04-28

**Authors:** Zuzana Míšková, Eva Lorencová, Richardos Nikolaos Salek, Tereza Koláčková, Ludmila Trávníková, Anita Rejdlová, Leona Buňková, František Buňka

**Affiliations:** 1 Department of Food Technology, Faculty of Technology, Tomas Bata University in Zlín, nám. T.G. Masaryka 5555, 760 01 Zlín, Czech Republic; miskova@utb.cz (Z.M.);; 2 Department of Environmental Protection Engineering, Faculty of Technology, Tomas Bata University in Zlín, nám. T.G. Masaryka 5555, 760 01 Zlín, Czech Republic; 3 Laboratory of Food Quality and Safety Research, Department of Logistics, Faculty of Military Leadership, University of Defence, Kounicova 65, 662 10 Brno, Czech Republic

**Keywords:** biogenic amines, wine, Central Europe, food safety, HPLC

## Abstract

The decarboxylation of the corresponding amino acids by microorganisms leads to the formation of biogenic amines (BAs). From a toxicological point of view, BAs can cause undesirable physiological effects in sensitive individuals, particularly if their metabolism is blocked or genetically altered. The current study aimed to monitor and evaluate the content of eight biogenic amines (BAs) in 232 samples of wines (white, rosé, red) produced in the Central European region (Zone B). White wines (180 samples), rosé wines (17 samples), and red wines (35 samples) were analyzed. High-performance liquid chromatography equipped with a ultraviolet–visible diode array detector (UV/VIS DAD) was applied to identify and quantify the BAs present in wines. In general, histamine (HIS), tyramine (TYM), putrescine (PUT), cadaverine (CAD), phenylethylamine (PEA), spermine (SPN) and spermidine (SPD) were detected in all tested wine samples. Tryptamine (TRM) was not present in any of the samples examined. In white and red wines, SPD, TYM, and PUT were most often detected. Regarding rosé wines, the three major BAs were SPN, TYM, and CAD. The BA content in red wines was generally higher than in rosé and white wines. However, HIS concentrations above the recommended limit of 10 mg/L were detected in 9% of the red wine samples. In addition, alarming levels of PUT, HIS, TYM, and PEA, with serious potential impact on consumer health, were recorded in two red wine samples. On the whole, the presence and concentrations of BAs in wine should be constantly evaluated, primarily because alcohol intensifies the hazardous effects of BAs.

## 1. Introduction

Biogenic amines (BAs) are biologically active organic bases of low molecular weight, and some of them (such as serotonin, histamine, and tyramine), are crucial to the physiology of humans, animals, and plants. BAs in food and beverages are produced primarily by the microbial decarboxylation of amino acids and by the amination of aldehydes and ketones [1,2,3,4]. Moreover, the human body can use a small amount of BAs as a precursor for the synthesis of hormones, alkaloids, nucleic acids, and proteins [1,2]. However, high concentrations of BAs in the diet may have direct toxic effects on humans [5,6]. BAs have the potential to be hazardous to consumers at high concentrations, with symptoms (e.g., hypo or hypertension, heart palpitations, headaches, migraines, respiratory disorders, gastrointestinal difficulties, skin allergies) that vary according to their type [7].

Furthermore, the effect of some BAs may be strengthened by the synergistic effect of other BAs [8]. Nonetheless, it is known that putrescine and cadaverine can promote the toxic effects of tyramine and histamine due to the inhabitation of human detoxifying mechanisms [9]. BAs are degraded by enzyme complexes, mainly by monoaminooxidases, diaminoxidases, and histidine-methyltransferases. Additionally, ethanol and acetaldehyde act as inhibitors of these three complexes, leading to an increased toxic effect of BAs. Therefore, it is important to analyze the BA content in alcoholic beverages, because alcohol is one of the factors that could alter the detoxification mechanism (by reducing the activity of diaminoxidase). Furthermore, certain BAs, including putrescine, cadaverine, spermidine, and spermine, may react with nitrite to form carcinogenic substances (nitrosamines) [1,2,10].

Wine is a beverage in which a significant amount of BAs are expected [2]. It is almost impossible to produce wine without the presence of BAs [11,12]. BAs enter wine from raw materials or are formed in wine during fermentation processes. These substances are mostly produced by microorganisms by means of decarboxylation of amino acids during alcoholic, and above all, malolactic, fermentation [13,14]. The main producers of BAs in wines are lactic acid bacteria (LAB, mainly representatives of the genera *Oenococcus*, *Lactobacillus*, *Pediococcus* and *Enterococcus*); in general, they are capable of producing histamine, tyramine, phenylethylamine and putrescine [15,16,17]. In several studies, the production of histamine, tyramine, and putrescine by LAB isolated from wine was examined, and it was found that this property is not determined by their genera, rather it is directly related to given bacterial strains [18,19,20,21,22]. Additionally, among the BAs detected in grapes (raw material), putrescine and spermidine predominate; their concentration depends mainly on the specific composition of the soil and the agrochemical and oenological technologies used [23,24]. The occurrence of BAs in wine is mainly determined by the decarboxylase activity of naturally occurring microorganisms, the starter microorganisms added during production, and the contaminating microflora. To ensure product safety, it is therefore important to adhere to good manufacturing and hygiene practices during primary production and postproduction processes [2,25,26,27].

Regulation 1308/2013 of the EU (Regulation (EU) No. 1308/2013 of the European Parliament and of the Council) [28], in establishing common organization of agricultural product markets, divides grapevine cultivation into the following winegrowing zones: A, B, CI, CII, CIIIa, and CIIIb, respectively. From the winegrowing zones CI, CII, CIIIa, and CIIIb (mainly southern European countries), the largest number of studies on the occurrence of BAs in wine are available [12,23,24,24,25,26,27,28,29,30,31,32,33,34,35,36]. However, very few such documents have focused on the presence of BAs in wines from Zone B (winegrowing zone), for example, from Central European countries [37,38]. Therefore, it has not yet been possible to assess the importance of the presence of BAs in wines produced in the Central European region over a sufficiently representative sample of wines, and evaluate it from a food safety point of view. Due to the fact that the European Union produces more wine than any other region in the world (the average annual production from 2014 to 2018 was 167 million hectoliters, accounting for 65% of the world’s wine production), sufficient production of high-quality and safe wines is essential for the local economy [39,40]. Moreover, knowledge of the BA profile (types and concentration) of wines is of great significance and for both consumers and producers; thus, this knowledge could serve as a blueprint for presenting important information for safety and quality control during the manufacturing, distribution, and storage of wines.

Given the significance of BAs in determining the quality of food and beverages, their presence in wine is a crucial factor of the oenology sector. In fact, a highly complicated set of variables determines the BAs’ final concentration in wines, which poses a threat to the product’s quality and safety [2]. The current study aimed to determine and evaluate the occurrence of eight BAs (histamine—HIS; tyramine—TYM; tryptamine—TRM; putrescine—PUT; cadaverine—CAD; phenylethylamine—PEA; spermine—SPN; and spermidine—SPD) in wines produced in the wine growing Zone B (Central Europe), and to assess the importance of their potential hazard in the context of food safety.

## 2. Materials and Methods

### 2.1. Wine Samples

In total, 232 wine samples (mainly monovarietal) from the Central European wine growing region (Zone B) were collected during the period of 2020–2022 from small- to medium-scale wineries. Of the total amount, 180 samples were white wines, 17 samples were rosé wines, and 35 samples were red wines. More specifically, there were 25 varieties (*Vitis vinifera* L.) of white wines, 9 varieties of rosé wines, and 13 varieties of red wines. The samples were collected in sterile glass bottles (volume 0.75 L). In addition, dry wines, semi-dry wines, semi-sweet wines, and sweet wines were examined.

pH values were determined in the wine samples using a pH meter (pH Spear Eutech—pH tester with fixed puncture electrode, Eutech Instruments, The Netherlands, Nijkerk). Measurements were repeated three times, and the measured values were then averaged.

### 2.2. Determination of Biogenic Amine Content

#### 2.2.1. Sample Treatment and Chromatographic Conditions for Biogenic Amine Analysis

Wine samples were diluted 1:1 (*v*/*v*) with perchloric acid (c = 1.2 mol/L). According to Komprda et al. [41], the acidified mixture was filtered (0.45 m), and the filtrate was then exposed to derivatization. Eight BAs (HIS, TYM, PUT, CAD, PEA, SPN, SPD, and TRM) were determined using high-performance liquid chromatography (LabAlliance, State College, New York, NY, USA; Agilent Technologies, Agilent, Paolo Alto, CA, USA) after derivatization with dansyl-chloride. The derivatized samples were filtered (0.22 μm) and applied on the column (ZORBAX Eclipse Plus C18, 50 mm × 3.0 mm, 1.8 μm; LabAlliance, State College, USA; Agilent Technologies, Agilent, Paolo Alto, Santa Clara, CA, USA) of a chromatographic system (pump and autosampler LabAlliance, State College, PA, USA); degasser, UV/VIS-DAD detector (λ = 254 nm) and column thermostat (Agilent Technologies, Agilent, Paolo Alto, CA, USA). The conditions for separation and detection of BA are described by Komprda et al. [41]; 1.7-heptanediamine (Sigma-Aldrich, St. Louis, MO, USA) was used as an internal standard. Each wine sample was analyzed from two different containers (of the same production batch), the samples from each container were derivatized three times, and each derivatized mixture was positioned onto the chromatographic column three times (3 derivatizations × 3 repetitions × 2 samples from each batch = 18; the total number of analyses of all 232 wines was 4176).

#### 2.2.2. Validation Process of the Method

According to Komprda et al. [41], the concentration of BAs in the sample was adjusted based on the method of internal standard (1,7-diaminoheptane), due to the multiple processes involved in sample preparation. The reproducibility, recovery, limits of detection and quantification were all determined as part of the method’s validation process. Moreover, by injecting five extracts of the selected wine sample with a low BA content and a mixture of the BAs standards after derivatization ten times, respectively, the repeatability of the analytical technique (expressed as a relative standard deviation; RSD) was examined. The values of RSD are shown for the apparatus and method repeatability, respectively. Recoveries were assessed by repeatedly (five times) adding a mixture of BAs standards with a concentration level of 2 mg/L to a real wine sample [41]. The recovery of individual BAs was in the range of 89.2–97.9%. The LOD (Table 1) and LOQ (Table 1) were determined according to standard chromatography procedures.

### 2.3. Statistical Analyses

Differences in the occurrence of BAs in individual samples were statistically evaluated using the Kruskal-Wallis and Wilcoxon tests. Correlation analysis was performed using the Spearman correlation coefficient. Unistat^®^ 5.6 (Unistat Ltd., London, UK) statistical software was used to process the data, employing a significance level of 0.05.

## 3. Results and Discussion

Table 1 presents the LOD, LOQ, recovery rates, linearity ranges and correlation coefficients. Regarding the LOD and LOQ, the BAs studied ranged from 0.013 to 1.128 mg/L and 0.15 to 0.28 mg/L, respectively. In addition, the recovery rates for the BAs ranged from 76.4% to 97.9%.

The pH values were measured in 232 samples of wine from the Central European winegrowing region (Zone B). The pH values for the white wines ranged from 2.08 to 3.94. In rosé wines, the pH values were measured in the range of 2.24–3.58; in red wines, they were from 2.14 to 3.88 (Table 2, Table 3 and Table 4). 

The results of the BA content of the individual analyzed wine samples are presented in Table 2, Table 3 and Table 4 (in particular, exact concentrations of the detected BAs are shown in Tables S1–S3 in the Supplementary Files). TRM did not appear in any of the wine samples examined. The results of the determination of the content of BAs in white wines are specified in Table 1. Generally, 98% of white wines contained BAs in the range of 0.1–34.6 mg/L. Most of the SPN (95% of samples) and TYM (92% of the samples) occurred in the white wines tested. In addition, PUT was determined in 39% of the samples, PEA in 27% of the samples, and CAD in 20% of the samples. Similarly, SPD (7% of the samples) and HIS (6% of the samples) were detected. However, a significant concentration of BAs (concentrations ≥ 10 mg/L) was recorded in white wines in 12% of the samples (*p* < 0.05); e.g., TYM, PUT, and SPN. The highest measured BA value (34.6 mg/L for TYM) was detected in the Pinot Blanc sample (*p* < 0.05). The second highest concentration was associated with PUT (30.4 mg/L) in the sample of the mixture of Moravian Muscat and Veltliner Green. In the “Děvín” grape variety, the highest amount of SPN determined was 12.9 mg/L. In 41.1% of the white wine samples, the BA content was in the range of 5–10 mg/L. The highest measured concentrations (≥10 mg/L) occurred in two wine samples, namely for SPN in Veltliner Green and for TYM in Tramini (*p* < 0.05). A BA level of 1–5 mg/L was present in 90% of the samples. In 49.4% of the white wine samples, only small amounts of BAs were detected (≤1 mg/L).

Furthermore, in white wine samples, the highest HIS content was detected in the Sauvignon Blanc sample (4.5 mg/L; *p* < 0.05). Regarding TYM, the highest reported value was 34.6 mg/L, in the Pinot Blanc sample (*p* < 0.05). Additionally, the highest amount of PEA was 6.5 mg/L, detected in the sample of Riesling Italico.

The results of the determination of the content of BAs in rosé wines are specified in Table 2. In general, all the rosé wines examined contained BAs in the range of 0.6 to 10.5 mg/L. The most abundant BA in rosé wines was SPN, which was detected in 88% of the samples. The second most frequent BA was TYM, which was detected in 77% of the samples. The other measured BAs were CAD (18% of samples), HIS, PEA (12% of samples), and PUT (6% of samples). SPD did not appear in any of the 17 samples of rosé wines. However, a concentration of BA above 10 mg/L was detected in only one sample of rosé wine (Andre; *p* < 0.05). In particular, SPN was present in this sample at a level of 10.5 mg/L. A level of 5–10 mg/L BA was represented in 53% of the rosé wine samples. A BA level of 1–5 mg/L was detected in 47% of rosé wines; a level of up to 1 mg/L was detected in 24% of the remaining samples.

The highest concentrations of HIS (2.9 mg/L) were detected in the sample of the mixture of Andre and Blaufränkisch varieties (*p* < 0.05). The highest amount of TYM in rosé wines was 9.3 mg/L, which was determined in the Zweigelt sample (*p* < 0.05). The highest concentration of PEA detected in the samples of rosé wine was 2.2 mg/L, namely in the sample of Merlot (*p* < 0.05).

The results of the determination of the content of BAs in red wines are specified in Table 3. 97% of red wines contained BAs in the range of 0.6–272.0 mg/L. As in the case of rosé and white wines, the most common BA was SPN, determined in 86% of red wine samples. The second most represented BA in red wines was TYM, which was contained in 80% of the samples. The third most common BA was PUT, occurring in 69% of red wine samples, of which 50% of the given sample quantity were determined to have significant PUT levels above 10 mg/L. Thus, although PUT was not the most frequently occurring BA, it was definitely the amine with the highest concentration values in the samples of red wines. Subsequently, HIS was detected in 37% of the tested samples, and SPD in 29% of the wine samples. The occurrence of CAD and PEA was almost identical (17% of the samples). In red wines, a significant number of BAs were detected (level greater than 10 mg/L) in 46% of the samples. This level included all BAs studied except CAD and SPD. The highest concentration value for PUT was detected in the sample of the Merlot variety (272.0 mg/L; *p* < 0.05). The sample of Merlot red wine mentioned above also contained significant amounts of HIS (19.4 mg/L; *p* < 0.05) and PEA (17.8 mg/L). Another sample with significant levels of BAs was that of Portugieser, in which 74.0 mg/L of PUT and 16.2 mg/L of TYM were determined. In general, in 83% of the red wine samples, BAs were present at a level of 5–10 mg/L. A level of 1–5 mg/L of BA was detected in 77% of the samples; a level up to 1 mg/L was detected in 9% of the red wine samples.

In samples of red wines from the Central European wine region, significant amounts of HIS were detected, even 19.4 mg/L in the Merlot sample. High concentrations of PEA were also measured, namely 17.8 mg/L in the Merlot sample. For TYM, the highest amount of 16.2 mg/L was detected in the sample of Portugieser.

The correlation analysis showed that the dependence of the content of BAs on the pH value of the wines was not significant (*p* ≥ 0.05). One of the few studies to discuss this is that of Comuzzo et al. [42]; however, in this study, it was determined that the higher the pH, the higher the BA content in the wine. In 2013, Comuzzo et al. [42] determined that a high pH value can cause greater bacterial growth and can result in significant concentrations of BAs in wine. However, the pH value is only one of the factors that influence the production of BAs. The ability of the present microflora to produce BAs as well as the presence of potentiators of this metabolism are key factors here [41]. Therefore, the total amount of BAs in wine is affected by many factors, such as the raw material itself, the amino acid composition after alcohol fermentation, the microflora present therein, the oenological processes utilized, etc. [1,2,11,12,23,43].

The results showed that the BA content was higher in the red wine samples than in the rosé and white wine samples (*p* < 0.05), which may be due to different production technologies and longer contact times of the pomaces with the extraction liquid (maceration process) during the production of red wines. Similarly, Comuzzo et al. [42], Ferreira et al. [28], and Leitao et al. [44] determined higher levels of BAs in red wines compared to white wines in their studies. Landete et al. [24] and Vidal-Carou et al. [45] also detected a higher concentration of BAs in red wines than those present in rosé and white wines.

Coton et al. [23] and Landete et al. [24] stated that the occurrence of PUT in wine is heavily influenced by the raw material; moreover, the low potassium content in the soil could increase the amount of this BA in the plant itself and thus also in the wine. In the work of Coton et al. [23], Landete et al. [24], and Lonvaud-Funel [21], TYM, PUT, and PEA were considered the main BAs in wine. Additionally, in the studies of Leitao et al. [44] and Comuzzo et al. [42], HIS, TYM, and PUT were identified as BAs that are the most represented in red wines. In white wines, Leitao et al. [44] specified that HIS, PUT, and CAD were the BAs most represented. Moreover, Landete et al. [24] stated that LAB are mainly responsible for the higher levels of HIS, TYM, and PEA. This was based on the results obtained, where white and rosé wines, in which malolactic fermentation occurred, contained similar amounts of BAs to those of red wines.

TRM was not detected in any sample of wines. Ferreira et al. [30], who also did not detect TRM in samples of Portuguese wines, also reached the same conclusion. In the study by Landete et al. [24], and Lonvaud-Funel [14], the level of TRM in wines was detected in very low concentrations compared to other BAs.

However, high concentrations of BAs in red wine samples can indicate failure to comply with proper hygiene and oenological practices during production [44].

The results obtained were compared with the toxic doses of BAs in alcoholic beverages proposed by Bodmer et al. [46], Halász et al. [47] and Lehtonen et al. [8], e.g., a concentration of 2–10 mg/L of HIS, 10–80 mg/L of TYR, and 3 mg/L of PHE. HIS concentrations greater than 10 mg/L were detected in 9% of red wine samples. TYM concentrations greater than 80 mg/L were not detected in any of the samples tested. Furthermore, PEA occurred at concentrations greater than 3 mg/L in 6% of the samples of white wines and 3% of the samples of red wines. Although most wines from the Central European wine region did not contain significantly high amounts of BA, it is necessary to consider the amount of food consumed and the total concentration of BA when evaluating the toxic effects of BAs [48]. 

Based on the observed results, the concentration of BA could seriously affect the health of consumers, especially in combination with the ethanol in wines (a potentiator of BAs’ impact). Therefore, monitoring of BAs’ incidence should be regularly carried out to improve food safety maintenance. Despite the toxicity of BAs being recognized, and their high content in some matrices (foods/beverages), international legislation has not yet placed restrictions on BAs. The regulations in force in European Union (EU) do not concern wines; EC regulation 2073/2005 (as well as its amendment EC 1019/2013) [49,50] sets the food safety criteria for HIS exclusively in fish products. Additionally, some EU countries (Germany, the Netherlands, Belgium, France) have established maximum permissible limits for HIS in wine (in the range of 2–10 mg/L); however, these restrictions (limits) are optional and may cause serious issues in commercial transactions. According to the European Food Safety Authority (EFSA), further research is needed on the toxicity and associated concentrations of HIS and TYR, as well as the related potentiating effects of PUT and CAD, which may provide new insight into the development of new safety criteria for HIS in fermented foods other than fish [7,51,52].

## 4. Conclusions

Biogenic amines were found in all wines studied from the Central Europe wine region (Zone B). In red and white wines, seven biogenic amines were detected (HIS, TYM, PUT, CAD, PEA, SPN, SPD); TRM was not present in any of these wines. In rosé wines, six BAs were recorded (HIS, TYM, PUT, CAD, PEA, SPN); SPD and TRM were not determined in any sample of the rosé wines. However, higher amounts of BAs were detected in red wines, compared to white and rosé wines. In all types of wines examined, there were up to 24% of samples exceeding the recommended limits for HIS, TYM, and PEA. Moreover, in two samples of red wines, there was an over-the-limit amount of HIS, TYM, PUT, and PEA, which may pose a health risk to sensitive individuals. It was also confirmed that red wines pose a greater risk from the point of view of food safety. With regard to the results obtained, the presence and amounts of BAs in wine should be continuously monitored, mainly because of the presence of alcohol, which increases the toxic effects of BAs.

## Figures and Tables

**Table 1:** Values of (mg/L) ^a^ limits of detection (LOD) and limits of quantification (LOQ) for biogenic amines monitored in wine samples produced in the Central Europe region.

Biogenic Amine	LOD (mg/L)	LOQ (mg/L)	Recovery Rates (%)	Linearity Ranges (mg/L)	R^2^
Histamine	0.102 ± 0.001	0.21 ± 0.01	97.5	0.1–200.0	0.9998
Tyramine	0.015 ± 0.001	0.18 ± 0.01	97.9	0.2–200.0	0.9996
Tryptamine	0.128 ± 0.001	0.61 ± 0.02	94.6	0.2–80.0	0.9997
Phenylalanine	0.058 ± 0.001	0.17 ± 0.01	96.9	0.1–50.0	0.9997
Putrescine	0.112 ± 0.001	0.28 ± 0.02	89.2	0.1–300.0	0.9998
Cadaverine	0.081 ± 0.001	0.19 ± 0.01	97.6	0.2–100.0	0.9997
Spermidine	0.019 ± 0.001	0.16 ± 0.01	77.8	0.2–40.0	0.9996
Spermine	0.013 ± 0.001	0.15 ± 0.01	76.4	0.1–40.0	0.9998

^a^ The results are expressed as mean ± standard deviation (*n* = 6).

**Table 2:** Biogenic amine content in white wines from the Central Europe region.

Wine	Number of Samples	pH	Biogenic Amine Content						
			(ND/+/++/+++/++++) ^a^						
			HIS	TYM	PUT	CAD	PEA	SPD	SPN
Aurelius	1	3.15 ± 0.02	1/0/0/0/0	0/0/1/0/0	1/0/0/0/0	1/0/0/0/0	1/0/0/0/0	1/0/0/0/0	0/0/0/1/0
Děvín	1	3.49 ± 0.03	1/0/0/0/0	0/0/1/0/0	1/0/0/0/0	1/0/0/0/0	1/0/0/0/0	1/0/0/0/0	0/0/0/0/1
Hibernal	1	3.29 ± 0.03	1/0/0/0/0	0/0/1/0/0	1/0/0/0/0	1/0/0/0/0	1/0/0/0/0	1/0/0/0/0	0/0/0/1/0
Chardonnay	14	3.20–3.72	13/1/0/0/0	0/5/6/2/1	9/1/2/2/0	12/2/0/0/0	10/3/0/1/0	13/1/0/0/0	0/1/8/5/0
Irssai Olivér	1	3.40 ± 0.01	1/0/0/0/0	0/0/0/1/0	0/0/0/0/1	1/0/0/0/0	1/0/0/0/0	1/0/0/0/0	0/0/0/1/0
Johanniter	1	3.16 ± 0.02	1/0/0/0/0	0/0/1/0/0	1/0/0/0/0	1/0/0/0/0	1/0/0/0/0	1/0/0/0/0	0/0/0/1/0
Müller Thurgau	10	2.99–3.94	9/0/1/0/0	0/0/9/1/0	7/0/1/1/1	8/0/2/0/0	10/0/0/0/0	9/0/1/0/0	0/0/2/7/1
Moravian Muscat	5	2.58–3.37	5/0/0/0/0	1/0/4/0/0	5/0/0/0/0	5/0/0/0/0	5/0/0/0/0	5/0/0/0/0	1/0/2/1/1
Mixture of Moravian Muscat and Veltliner Green	2	2.49–3.34	2/0/0/0/0	1/0/1/0/0	1/0/0/0/1	2/0/0/0/0	2/0/0/0/0	2/0/0/0/0	1/0/0/1/0
Muscat Ottonel	1	3.84 ± 0.01	0/0/1/0/0	0/0/1/0/0	0/0/0/0/1	1/0/0/0/0	0/0/1/0/0	1/0/0/0/0	0/0/0/1/0
Neuburger	5	2.08–3.28	5/0/0/0/0	1/0/3/1/0	5/0/0/0/0	4/0/1/0/0	3/0/2/0/0	4/1/0/0/0	1/0/1/3/0
Mixture of Neuburg and Veltliner Green	1	2.57 ± 0.01	1/0/0/0/0	0/0/1/0/0	1/0/0/0/0	0/1/0/0/0	1/0/0/0/0	1/0/0/0/0	0/0/0/1/0
Pálava	3	2.36–3.38	3/0/0/0/0	0/0/2/1/0	2/0/0/0/1	2/0/1/0/0	2/0/1/0/0	3/0/0/0/0	1/0/0/2/0
Pinot Blanc	8	2.75–3.56	8/0/0/0/0	1/2/4/0/1	4/0/3/1/0	5/3/0/0/0	4/2/2/0/0	8/0/0/0/0	1/0/6/1/0
Mixture of Pinot Blanc and Chardonnay	1	2.52 ± 0.03	0/1/0/0/0	0/0/0/0/1	1/0/0/0/0	1/0/0/0/0	0/0/1/0/0	1/0/0/0/0	0/0/1/0/0
Pinot Gris	19	3.22–3.88	17/0/2/0/0	1/4/11/2/1	10/1/6/1/1	14/4/1/0/0	13/3/2/1/0	17/0/2/0/0	0/0/13/6/0
Riesling, Weisser	22	2.08–3.87	21/0/1/0/0	1/5/12/3/1	14/1/6/1/0	17/3/2/0/0	17/4/1/0/0	22/0/0/0/0	1/0/11/9/1
Riesling Italico	12	2.31–3.69	10/1/1/0/0	0/2/10/0/0	6/0/5/1/0	9/1/2/0/0	9/0/2/1/0	11/0/1/0/0	0/0/10/0/2
Sauvignon Blanc	26	2.43–3.74	25/0/1/0/0	3/11/8/3/1	16/0/9/0/1	21/4/1/0/0	18/4/3/1/0	24/1/1/0/0	1/0/19/6/0
Mixture of Sauvignon Blanc and Tramini	1	2.24 ± 0.02	1/0/0/0/0	0/0/1/0/0	1/0/0/0/0	0/1/0/0/0	1/0/0/0/0	1/0/0/0/0	0/0/1/0/0
Solaris	1	2.89 ± 0.01	1/0/0/0/0	1/0/0/0/0	1/0/0/0/0	1/0/0/0/0	1/0/0/0/0	1/0/0/0/0	0/0/1/0/0
Sylvaner	2	3.13–3.24	2/0/0/0/0	0/0/2/0/0	2/0/0/0/0	2/0/0/0/0	2/0/0/0/0	1/0/1/0/0	0/0/1/1/0
Tramini	14	3.12–3.71	14/0/0/0/0	0/7/5/2/0	6/0/5/3/0	12/2/0/0/0	10/0/4/0/0	14/0/0/0/0	0/0/10/3/1
Mixture of Veltliner Red and Riesling Italico	1	2.20 ± 0.02	1/0/0/0/0	0/0/1/0/0	1/0/0/0/0	1/0/0/0/0	1/0/0/0/0	1/0/0/0/0	0/0/0/1/0
Veltliner Green	27	2.65–3.75	27/0/0/0/0	4/9/10/2/2	14/1/10/2/0	22/4/1/0/0	18/3/6/0/0	24/2/1/0/0	2/0/15/9/1

^a^ Biogenic amine contents (*n* = 10) were expressed using intervals as follows: “ND” not detected, “+” 0–1 mg/L, “++” 1–5 mg/L, “+++” 5–10 mg/L, “++++” <40 mg/L.

**Table 3:** Biogenic amine content in rosé wines from the Central Europe region.

Wine	Number of Samples	pH	Biogenic Amine Content						
			(ND/+/++/+++/++++) ^a^						
			HIS	TYM	PUT	CAD	PEA	SPD	SPN
André	1	3.58 ± 0.01	1/0/0/0/0	0/0/0/1/0	1/0/0/0/0	1/0/0/0/0	1/0/0/0/0	1/0/0/0/0	0/0/0/0/1
Mixture of André and Blaufränkisch	1	2.40 ± 0.01	0/0/1/0/0	0/0/1/0/0	1/0/0/0/0	1/0/0/0/0	1/0/0/0/0	1/0/0/0/0	0/0/0/1/0
Cabernet Sauvignon	1	2.57 ± 0.03	1/0/0/0/0	1/0/0/0/0	1/0/0/0/0	0/1/0/0/0	1/0/0/0/0	1/0/0/0/0	1/0/0/0/0
Blaufränkisch	6	2.24–3.43	6/0/0/0/0	1/0/3/2/0	5/0/0/1/0	4/2/0/0/0	5/0/1/0/0	6/0/0/0/0	1/0/0/5/0
Merlot	1	2.54 ± 0.02	1/0/0/0/0	0/0/1/0/0	1/0/0/0/0	1/0/0/0/0	0/0/1/0/0	1/0/0/0/0	0/0/0/1/0
Portugieser and Blauer	1	2.96 ± 0.03	1/0/0/0/0	1/0/0/0/0	1/0/0/0/0	1/0/0/0/0	1/0/0/0/0	1/0/0/0/0	0/0/1/0/0
Pinot noir	2	2.68–3.29	1/0/1/0/0	0/0/2/0/0	2/0/0/0/0	2/0/0/0/0	2/0/0/0/0	2/0/0/0/0	0/0/2/0/0
Saint Laurent	1	3.22 ± 0.02	1/0/0/0/0	0/1/0/0/0	1/0/0/0/0	1/0/0/0/0	1/0/0/0/0	1/0/0/0/0	0/0/0/1/0
Zweigelt	3	3.03–3.22	3/0/0/0/0	1/0/1/1/0	3/0/0/0/0	3/0/0/0/0	3/0/0/0/0	3/0/0/0/0	0/0/2/1/0

^a^ Biogenic amine contents (*n* = 10) were expressed using intervals as follows: “ND” not detected, “+” 0–1 mg/L, “++” 1–5 mg/L, “+++” 5–10 mg/L, “++++” <20 mg/L.

**Table 4:** Biogenic amine content in red wines from the Central Europe region.

Wine	Number of Samples	pH	Biogenic Amine Content						
			(ND/+/++/+++/++++) ^a^						
			HIS	TYM	PUT	CAD	PEA	SPD	SPN
Alibernet	1	2.38 ± 0.02	0/0/1/0/0	0/0/1/0/0	0/0/0/1/0	1/0/0/0/0	1/0/0/0/0	1/0/0/0/0	0/0/1/0/0
Mixture of Alibernet and Rubinet	1	2.91 ± 0.03	0/0/0/0/1	0/0/0/1/0	0/0/0/0/1	1/0/0/0/0	1/0/0/0/0	1/0/0/0/0	0/0/1/0/0
André	4	2.14–3.59	4/0/0/0/0	0/0/3/1/0	3/0/0/1/0	3/1/0/0/0	3/0/1/0/0	3/0/1/0/0	0/0/1/2/1
Cabernet Sauvignon	3	3.12–3.55	2/0/0/1/0	1/0/2/0/0	1/0/0/2/0	3/0/0/0/0	2/0/1/0/0	2/1/0/0/0	1/0/1/1/0
Mixture of Cabernet Moravia and Alibernet	1	2.78 ± 0.02	0/0/1/0/0	0/0/0/1/0	0/0/0/0/1	1/0/0/0/0	0/0/1/0/0	1/0/0/0/0	0/0/0/1/0
Dornfelder	2	3.28–3.68	2/0/0/0/0	0/0/2/0/0	1/0/0/1/0	1/0/1/0/0	2/0/0/0/0	1/0/1/0/0	0/0/0/2/0
Mixture of Blaufränkisch and Saint Laurent and Portugieser, Blauer	1	2.89 ± 0.02	0/0/0/1/0	0/0/0/1/0	0/0/0/0/1	1/0/0/0/0	1/0/0/0/0	1/0/0/0/0	1/0/0/0/0
Blaufränkisch	5	2.53–3.54	2/0/3/0/0	1/0/2/1/1	1/0/0/0/4	3/1/1/0/0	3/0/2/0/0	2/1/2/0/0	0/0/2/3/0
Merlot	3	2.90–3.52	1/0/1/0/1	1/0/0/2/0	0/0/0/0/3	3/0/0/0/0	2/0/0/0/1	2/0/0/1/0	2/0/0/1/0
Portugieser and Blauer	6	2.99–3.56	5/0/0/0/1	2/0/3/0/1	2/0/0/2/2	4/0/2/0/0	6/0/0/0/0	5/0/1/0/0	1/0/2/2/1
Pinot noir	2	3.76–3.88	2/0/0/0/0	0/0/1/0/1	1/0/0/1/0	2/0/0/0/0	2/0/0/0/0	1/0/1/0/0	0/0/0/2/0
Saint Laurent	5	3.11–3.76	3/0/2/0/0	2/0/2/0/1	2/0/0/3/0	5/0/0/0/0	5/0/0/0/0	4/0/0/1/0	0/0/2/3/0
Zweigelt	1	3.20 ± 0.02	1/0/0/0/0	0/0/1/0/0	0/0/0/1/0	1/0/0/0/0	1/0/0/0/0	1/0/0/0/0	0/0/0/1/0

^a^ Biogenic amine contents (*n* = 10) were expressed using intervals as follows: “ND” not detected, “+” 0–1 mg/L, “++” 1–5 mg/L, “+++” 5–10 mg/L, “++++” <300 mg/L.

## Data Availability

The data presented in this study are available on request from the corresponding author.

## Supplementary Materials

The following supporting information can be downloaded at: https://www.mdpi.com/article/10.3390/foods12091835/s1, Table S1: Biogenic amines content (mg/L) ^a^ in white wines from the Central European region, Table S2: Biogenic amines content (mg/L) ^a^ in rosé wines from the Central European region, Table S3: Biogenic amines content (mg/L) ^a^ in red wines from the Central European region.
