# Supplementary material for: Occurrence of Biogenic Amines in Wines from the Central European Region (Zone B) and Evaluation of Their Safety
Source: Foods. 2023 Apr 28;12(9):1835. doi: 10.3390/foods12091835 (PMC10178851; doi:10.3390/foods12091835)
Supplement: Supplementary file 1 [file foods-12-01835-s001.zip › foods-2318220-supplementary.pdf]

Table S1. Biogenic amines content (mg/L)[a] in white wines from the Central European region.

Number	Wine	Biogenic amines content **						
		HIS	TYM	PUT	CAD	PEA	SPD	SPN
1	Aurelius	ND *	2.4a±0.1	ND	ND	ND	ND	5.4b±0.3
2	Děvín	ND	4.1a±0.2	ND	ND	ND	ND	12.9b±0.6
3	Hibernal	ND	1.9a±0.1	ND	ND	ND	ND	5.1b±0.2
4	Chardonnay	ND	6.7a±0.3	7.4a±0.4	ND	ND	ND	6.8a±0.3
5	Chardonnay	ND	3.3a±0.2	ND	ND	ND	ND	7.9b±0.4
6	Chardonnay	0.7a±0.0	2.8b±0.2	ND	ND	ND	ND	6.8c±0.3
7	Chardonnay	ND	1.7a±0.1	ND	ND	ND	ND	5.1b±0.3
8	Chardonnay	ND	2.5a±0.1	5.7b±0.2	0.4c±0.1	ND	0.1d±0.1	3.8e±0.1
9	Chardonnay	ND	5.5a±0.2	3.9b±0.2	ND	0.7c±0.1	ND	4.4d±0.2
10	Chardonnay	ND	1.8a±0.1	1.3b±0.1	ND	0.5c±0.1	ND	2.8d±0.1
11	Chardonnay	ND	16.4a±0.8	0.9b±0.1	0.4c±0.1	0.2c±0.1	ND	0.9b±0.1
12	Chardonnay	ND	3.5a±0.2	ND	ND	ND	ND	3.3a±0.1
13	Chardonnay	ND	0.7a±0.1	ND	ND	ND	ND	3.5b±0.1
14	Chardonnay	ND	0.2a±0.1	ND	ND	ND	ND	3.5b±0.1
15	Chardonnay	ND	0.2a±0.1	ND	ND	ND	ND	2.5b±0.1
16	Chardonnay	ND	0.1a±0.1	ND	ND	ND	ND	1.5b±0.1
17	Chardonnay	ND	0.5a±0.1	ND	ND	5.1b±0.2	ND	5.3b±0.3
18	Irssai Olivér	ND	8.2a±0.3	10.1b±0.4	ND	ND	ND	5.4c±0.3
19	Johanniter	ND	1.8a±0.1	ND	ND	ND	ND	5.6b±0.3
20	Neuburg and Veltliner Green	ND	3.2a±0.2	ND	1.0b±0.1	ND	ND	5.3c ± 0.2
21	Pinot Blanc and Chardonnay	1.0a±0.1	22.8b±1.2	ND	ND	1.4c±0.1	ND	4.3d±0.2
22	Sauvignon Blanc and Tramini	ND	2.0a±0.1	ND	0.7b±0.1	ND	ND	4.6c±0.2
23	Veltliner Red and Riesling Italico	ND	3.7a±0.2	ND	ND	ND	ND	7.4b±0.3

24	Moravian Muscat and Veltliner Green	ND	2.4a±0.1	30.4b±1.5	ND	ND	ND	ND
25	Moravian Muscat and Veltiner Green	ND	ND	ND	ND	ND	ND	7.3a±0.3
26	Moravian Muscat	ND	ND	ND	ND	ND	ND	2.4a±0.1
27	Moravian Muscat	ND	2.1a±0.1	ND	ND	ND	ND	3.4b±0.1
28	Moravian Muscat	ND	4.1a±0.2	ND	ND	ND	ND	ND
29	Moravian Muscat	ND	2.4a±0.1	ND	ND	ND	ND	5.9b±0.3
30	Moravian Muscat	ND	2.9a±0.1	ND	ND	ND	ND	11.0b±0.4
31	Muller Thurgau	ND	1.8a±0.1	ND	ND	ND	ND	8.1b±0.4
32	Muller Thurgau	ND	3.7a±0.2	6.1b±0.3	ND	ND	ND	5.4c±0.2
33	Muller Thurgau	ND	1.3a±0.1	1.7b±0.1	ND	ND	1.3a±0.1	3.9c±0.2
34	Muller Thurgau	ND	3.9a±0.2	ND	ND	ND	ND	8.4b±0.4
35	Muller Thurgau	ND	2.4a±0.1	ND	ND	ND	ND	5.8b±0.3
36	Muller Thurgau	ND	1.6a±0.1	ND	1.3b±0.1	ND	ND	5.5c±0.3
37	Muller Thurgau	1.8a±0.1	7.0b±0.4	12.7c±0.6	1.1d±0.1	ND	ND	4.3e±0.2
38	Muller Thurgau	ND	2.7a±0.1	ND	ND	ND	ND	11.9b±0.6
39	Muller Thurgau	ND	2.1a±0.1	ND	ND	ND	ND	6.0b±0.3
40	Muller Thurgau	ND	3.0a±0.2	ND	ND	ND	ND	7.4b±0.4
41	Muscat Ottonel	2.8a±0.2	1.7b±0.1	23.2c±1.0	ND	2.3d±0.1	ND	6.2e±0.3
42	Neuburger	ND	ND	ND	ND	ND	1.0a±0.1	3.9b±0.2
43	Neuburger	ND	1.7a±0.1	ND	ND	ND	ND	ND
44	Neuburger	ND	6.2a±0.3	ND	ND	2.9b±0.1	ND	6.8a±0.4
45	Neuburger	ND	2.2a±0.1	ND	3.1b±0.2	3.1b±0.2	ND	6.1c±0.3
46	Neuburger	ND	1.8a±0.1	ND	ND	ND	ND	5.7b±0.3
47	Pálava	ND	5.2a±0.3	10.2b±0.4	1.7c±0.1	ND	ND	5.3a ± 0.2
48	Pálava	ND	4.7a±0.2	ND	ND	2.5b±0.1	ND	ND
49	Pálava	ND	4.3a ±0.2	ND	ND	ND	ND	8.2b ± 0.4
50	Pinot Blanc	ND	ND	ND	ND	ND	ND	6.2a±0.3
51	Pinot Blanc	ND	34.6a±1.8	ND	ND	ND	ND	ND
52	Pinot Blanc	ND	3.2a±0.1	2.7b±0.1	0.8c±0.1	1.2d±0.1	ND	4.7e±0.2
53	Pinot Blanc	ND	2.4a±0.1	1.6b±0.1	0.8c±0.1	0.3d±0.1	ND	4.1e±0.2

54	Pinot Blanc	ND	1.3a±0.1	1.3a±0.1	0.6b±0.1	0.1c±0.1	ND	1.7d±0.1
55	Pinot Blanc	ND	1.2a±0.1	5.1b±0.2	ND	3.7c±0.1	ND	4.9b±0.2
56	Pinot Blanc	ND	0.2a±0.1	ND	ND	ND	ND	3.3b±0.1
57	Pinot Blanc	ND	0.2a±0.1	ND	ND	ND	ND	3.4b±0.1
58	Pinot Gris	2.7a±0.2	8.3b±0.3	9.1b±0.5	ND	ND	ND	4.1c±0.2
59	Pinot Gris	ND	3.6a±0.2	4.4b±0.2	1.9c±0.1	ND	1.4d±0.1	7.9e±0.4
60	Pinot Gris	2.3a±0.1	7.3b±0.3	16.2c±0.9	ND	ND	ND	5.0d±0.3
61	Pinot Gris	ND	3.5a±0.2	ND	ND	ND	ND	7.2b±0.3
62	Pinot Gris	ND	1.4a±0.1	ND	ND	ND	ND	6.2b±0.3
63	Pinot Gris	ND	ND	3.8a±0.2	0.4b±0.0	ND	1.7c±0.1	3.6a±0.1
64	Pinot Gris	ND	2.8a±0.1	ND	ND	1.2b±0.1	ND	2.8a±0.1
65	Pinot Gris	ND	1.6a±0.1	1.9b±0.1	ND	0.3c±0.1	ND	4.5d±0.2
66	Pinot Gris	ND	1.4a±0.1	0.7b±0.1	0.8b±0.1	0.7b±0.1	ND	1.5a±0.1
67	Pinot Gris	ND	15.8a±0.7	1.9b±0.1	0.4c±0.1	0.7d±0.1	ND	5.8e±0.3
68	Pinot Gris	ND	1.2a±0.1	1.4a±0.1	0.4b±0.1	ND	ND	1.8c±0.1
69	Pinot Gris	ND	2.6a±0.1	2.0b±0.1	ND	ND	ND	2.7a±0.1
70	Pinot Gris	ND	0.1a±0.1	ND	ND	ND	ND	1.7b±0.1
71	Pinot Gris	ND	0.1a±0.1	ND	ND	ND	ND	2.1b±0.1
72	Pinot Gris	ND	0.1a±0.1	ND	ND	ND	ND	3.4b±0.1
73	Pinot Gris	ND	1.2a±0.1	ND	ND	ND	ND	5.3b±0.2
74	Pinot Gris	ND	0.7a±0.1	ND	ND	ND	ND	2.5b±0.1
75	Pinot Gris	ND	1.5a±0.1	ND	ND	4.4b±0.2	ND	3.7c±0.1
76	Pinot Gris	ND	2.2a±0.1	ND	ND	5.9b±0.3	ND	6.4b±0.2
77	Riesling Italico	ND	1.4a±0.1	4.7b±0.3	ND	ND	1.7c±0.1	4.9a±0.3
78	Riesling Italico	0.9a±0.1	3.0b±0.1	6.3c±0.4	1.1a± 0.1	ND	ND	4.2d±0.2
79	Riesling Italico	ND	3.8a±0.2	ND	ND	ND	ND	10.3b±0.5
80	Riesling Italico	ND	4.3a±0.2	ND	ND	ND	ND	10.1b±0.5
81	Riesling Italico	ND	1.5a±0.1	2.5b±0.1	ND	ND	ND	4.9c±0.3
82	Riesling Italico	ND	3.3a±0.2	ND	ND	ND	ND	4.7b±0.3
83	Riesling Italico	ND	3.4a±0.1	4.4b±0.2	ND	ND	ND	2.2c±0.1
84	Riesling Italico	ND	1.0a±0.1	1.9b±0.1	0.5c±0.1	ND	ND	1.9b±0.1
85	Riesling Italico	ND	3.1a±0.1	1.1b±0.1	1.3c±0.1	ND	ND	3.5d±0.1

86	Riesling Italico	ND	0.2a±0.1	ND	ND	1.8b±0.1	ND	1.6b±0.1
87	Riesling Italico	1.8a±0.1	0.9b±0.1	ND	ND	1.7a±0.1	ND	2.3c±0.1
88	Riesling Italico	ND	2.4a±0.1	ND	ND	6.4b±0.3	ND	4.9c±0.2
89	Riesling Weisser	ND	4.8a±0.2	ND	ND	ND	ND	3.9b±0.2
90	Riesling Weisser	ND	2.5a±0.1	ND	ND	ND	ND	8.6b±0.4
91	Riesling Weisser	ND	1.9a±0.1	ND	ND	ND	ND	5.3b±0.3
92	Riesling Weisser	ND	3.6a±0.2	ND	ND	ND	ND	6.6b±0.4
93	Riesling Weisser	1.7a±0.1	9.6b±0.5	9.7b±0.5	1.1c±0.1	ND	ND	6.1d±0.3
94	Riesling Weisser	ND	12.7a±0.6	ND	ND	ND	ND	10.8b±0.5
95	Riesling Weisser	ND	2.2a±0.1	ND	ND	ND	ND	8.1b±0.4
96	Riesling Weisser	ND	3.1a±0.1	ND	ND	ND	ND	7.7b±0.4
97	Riesling Weisser	ND	7.6a±0.3	1.6b±0.1	1.5b±0.1	0.5c±0.1	ND	4.3d±0.2
98	Riesling Weisser	ND	5.4a±0.2	2.5b±0.1	ND	ND	ND	4.2c±0.2
99	Riesling Weisser	ND	4.1a±0.2	1.1b±0.1	0.5c±0.1	0.6c±0.1	ND	1.8d±0.1
100	Riesling Weisser	ND	2.7a±0.1	0.6b±0.1	0.7b±0.1	ND	ND	2.2c±0.1
101	Riesling Weisser	ND	1.8a±0.1	3.6b±0.1	0.5c±0.1	0.5c±0.1	ND	3.6b±0.2
102	Riesling Weisser	ND	3.5a±0.2	1.6b±0.1	ND	0.6c±0.1	ND	1.7b±0.1
103	Riesling Weisser	ND	2.6a±0.1	ND	ND	ND	ND	2.4a±0.1
104	Riesling Weisser	ND	1.3a±0.1	3.5b±0.1	ND	ND	ND	5.9c±0.2
105	Riesling Weisser	ND	ND	ND	ND	ND	ND	ND
106	Riesling Weisser	ND	0.2a±0.1	ND	ND	ND	ND	4.4b±0.2
107	Riesling Weisser	ND	0.3a0.1	ND	ND	ND	ND	5.8b±0.3
108	Riesling Weisser	ND	0.2a±0.1	ND	ND	ND	ND	1.7b±0.1
109	Riesling Weisser	ND	0.5a±0.1	ND	ND	2.8b±0.1	ND	2.9b±0.1
110	Riesling Weisser	ND	0.4a±0.1	ND	ND	ND	ND	5.9b±0.3
111	Sauvignon Blanc	4.5a±0.2	11.0b±0.5	11.3b±0.6	ND	1.5c±0.1	ND	6.6d±0.3
112	Sauvignon Blanc	ND	ND	ND	ND	ND	ND	2.0a±0.1
113	Sauvignon Blanc	ND	ND	2.8a±0.2	ND	ND	1.0b±0.1	2.5a±0.1
114	Sauvignon Blanc	ND	2.7a±0.1	ND	ND	ND	ND	ND
115	Sauvignon Blanc	ND	1.2a±0.1	ND	1.1a±0.1	ND	ND	4.3b±0.2
116	Sauvignon Blanc	ND	2.6a±0.1	ND	ND	ND	ND	5.4b±0.2
117	Sauvignon Blanc	ND	ND	2.1a±0.1	0.9b±0.1	ND	3.4c±0.1	2.5d±0.1

118	Sauvignon Blanc	ND	2.5a±0.1	2.0b±0.1	ND	ND	ND	4.8c±0.2
119	Sauvignon Blanc	ND	6.6a±0.3	3.7b±0.1	0.8c±0.1	0.6c±0.1	ND	5.4d±0.2
120	Sauvignon Blanc	ND	1.8a±0.1	1.6a±0.1	ND	0.5b±0.1	ND	3.4c±0.2
121	Sauvignon Blanc	ND	2.6a±0.1	2.6a±0.1	0.5b±0.1	1.0c±0.1	ND	5.3d±0.2
122	Sauvignon Blanc	ND	6.9a±0.3	1.5b±0.1	0.4c±0.1	0.2c±0.1	ND	1.7b±0.1
123	Sauvignon Blanc	ND	2.4a±0.1	ND	ND	ND	ND	4.7b±0.2
124	Sauvignon Blanc	ND	2.8a±0.1	1.7b±0.1	ND	ND	ND	3.3c±0.1
125	Sauvignon Blanc	ND	8.8a±0.4	1.7b±0.1	ND	1.4c±0.1	ND	2.4d±0.1
126	Sauvignon Blanc	ND	0.9a±0.1	ND	ND	ND	ND	2.8b±0.1
127	Sauvignon Blanc	ND	0.6a±0.1	ND	ND	ND	ND	3.2b±0.1
128	Sauvignon Blanc	ND	0.2a±0.1	ND	ND	0.8b±0.1	ND	1.1c±0.1
129	Sauvignon Blanc	ND	0.1a±0.1	ND	ND	ND	ND	1.2b±0.1
130	Sauvignon Blanc	ND	0.3a±0.1	ND	ND	ND	ND	3.8b±0.2
131	Sauvignon Blanc	ND	0.1a±0.1	ND	ND	ND	ND	1.3b±0.1
132	Sauvignon Blanc	ND	0.1a±0.1	ND	ND	ND	ND	2.3b±0.1
133	Sauvignon Blanc	ND	0.4a±0.1	ND	ND	ND	ND	1.9b±0.1
134	Sauvignon Blanc	ND	0.1a±0.1	ND	ND	ND	ND	2.2b±0.1
135	Sauvignon Blanc	ND	0.6a±0.1	ND	ND	6.3b±0.2	ND	6.9c±0.3
136	Sauvignon Blanc	ND	0.6a±0.1	ND	ND	ND	ND	5.9b±0.3
137	Solaris	ND	ND	ND	ND	ND	ND	4.1±0.2
138	Sylvaner	ND	3.7a±0.2	ND	ND	ND	1.1b±0.0	3.2c±0.1
139	Sylvaner	ND	3.2a±0.2	ND	ND	ND	ND	6.7b±0.3
140	Tramini	ND	2.8a±0.1	5.6b±0.3	ND	ND	ND	4.3c±0.3
141	Tramini	ND	9.2a±0.4	5.1b±0.2	ND	1.5c±0.1	ND	7.9d±0.4
142	Tramini	ND	10.0a±0.5	9.4a±0.5	ND	3.9b±0.2	ND	9.2a±0.5
143	Tramini	ND	3.8a±0.2	ND	ND	ND	ND	11.9b±0.6
144	Tramini	ND	1.6a±0.1	3.6b±0.16	0.7c±0.1	ND	ND	2.7d±0.1
145	Tramini	ND	0.6a±0.1	2.2b±0.11	0.6a±0.1	ND	ND	2.2b±0.1
146	Tramini	ND	3.1a±0.1	ND	ND	ND	ND	1.9b±0.1
147	Tramini	ND	0.6a±0.1	4.8b±0.25	ND	ND	ND	2.1c±0.1
148	Tramini	ND	2.0a±0.1	3.8b±0.20	ND	1.0c±0.1	ND	2.9d±0.1
149	Tramini	ND	0.5a±0.1	2.2b±0.14	ND	ND	ND	4.2c±0.2

150	Tramini	ND	0.2a±0.1	ND	ND	ND	ND	2.6±0.1
151	Tramini	ND	0.8a±0.1	ND	ND	ND	ND	1.4±0.1
152	Tramini	ND	0.6a±0.1	ND	ND	2.1b±0.1	ND	3.7c±0.2
153	Tramini	ND	0.4a±0.1	ND	ND	ND	ND	7.3b±0.3
154	Veltliner Green	ND	1.7a±0.1	4.1b±0.2	ND	ND	ND	5.8c±0.3
155	Veltliner Green	ND	ND	ND	ND	ND	1.3a±0.1	3.4b±0.2
156	Veltliner Green	ND	3.4a±0.2	5.8b±0.3	ND	ND	ND	5.7b±0.2
157	Veltliner Green	ND	2.5a±0.1	ND	ND	ND	ND	7.9b±0.5
158	Veltliner Green	ND	1.8a±0.1	ND	ND	ND	ND	5.5b±0.3
159	Veltliner Green	ND	6.7a±0.3	9.2b±0.5	ND	ND	ND	10.0c±0.5
160	Veltliner Green	ND	4.2a±0.2	ND	1.5b±0.1	ND	ND	11.2c±0.7
161	Veltliner Green	ND	ND	ND	ND	ND	0.7a±0.0	2.0b±0.1
162	Veltliner Green	ND	1.5a±0.1	2.2b±0.1	0.3c±0.1	1.0d±0.1	0.4c±0.1	3.0e±0.1
163	Veltliner Green	ND	5.4a±0.2	2.4b±0.1	ND	ND	ND	5.9c±0.2
164	Veltliner Green	ND	1.6a±0.1	1.3b±0.1	ND	0.6c±0.1	ND	1.1b±0.1
165	Veltliner Green	ND	1.4a±0.1	1.7b±0.1	0.4c±0.1	ND	ND	4.4d±0.2
166	Veltliner Green	ND	0.9a±0.1	0.9a±0.1	0.7a±0.1	1.2b±0.1	ND	1.9c±0.1
167	Veltliner Green	ND	0.5a±0.1	4.1b±0.1	ND	ND	ND	1.9c±0.1
168	Veltliner Green	ND	1.4a±0.1	1.4a±0.1	ND	ND	ND	4.8b±0.2
169	Veltliner Green	ND	12.3a±0.6	1.3b±0.1	ND	0.6c±0.1	ND	2.6d±0.1
170	Veltliner Green	ND	10.5a±0.5	1.7b±0.1	0.4c±0.1	0.8d±0.1	ND	2.4e±0.1
171	Veltliner Green	ND	0.6a±0.1	ND	ND	ND	ND	2.6b±0.1
172	Veltliner Green	ND	0.5a±0.1	3.2b±0.1	ND	2.5c±0.1	ND	6.6d±0.3
173	Veltliner Green	ND	0.1a±0.1	ND	ND	ND	ND	1.0b±0.1
174	Veltliner Green	ND	ND	ND	ND	ND	ND	ND
175	Veltliner Green	ND	ND	ND	ND	ND	ND	ND
176	Veltliner Green	ND	0.1a±0.1	ND	ND	ND	ND	2.0b±0.1
177	Veltliner Green	ND	0.6a±0.1	ND	ND	ND	ND	1.1b±0.1
178	Veltliner Green	ND	1.5a±0.1	ND	ND	1.4a±0.1	ND	6.3b±0.3
179	Veltliner Green	ND	0.7a±0.1	ND	ND	3.0b±0.1	ND	5.9c±0.2
180	Veltliner Green	ND	0.4a±0.1	ND	ND	3.7b±0.1	ND	3.1c±0.1

* ND – not detected

** HIS: histamine; TYR: tyramine; PUT: putrescine; CAD: cadaverine; PHE: phenylethylamine; SPD: spermidine; SPM: spermine
[a] The values are expressed as mean ± standard deviation. The mean values within a line (the difference between individual BA) followed by different letters differ ($p < 0.05$).

Table S2. Biogenic amines content (mg/L)[a] in rosé wines from the Central European region.

Number	Wine	Biogenic amines content**						
		**HIS**	**TYM**	**PUT**	**CAD**	**PEA**	**SPD**	**SPN**
1	André	ND*	5.5a±0.3	ND	ND	ND	ND	10.5b±0.5
2	Blaufränkisch	ND	8.5a±0.4	5.5b±0.3	ND	ND	ND	6.3c±0.3
3	Blaufränkisch	ND	3.9a±0.1	ND	ND	ND	ND	8.9b±0.5
4	Blaufränkisch	ND	ND	ND	0.6a±0.1	ND	ND	ND
5	Blaufränkisch	ND	3.6a±0.2	ND	0.7b±0.1	1.1c±0.0	ND	6.1d±0.3
6	Blaufränkisch	ND	5.3a±0.2	ND	ND	ND	ND	5.8b±0.3
7	Blaufränkisch	ND	1.5a±0.1	ND	ND	ND	ND	5.6b±0.3
8	Cabernet Sauvignon	ND	ND	ND	0.7a±0.1	ND	ND	ND
9	Merlot	ND	3.1a±0.2	ND	ND	2.2b±0.1	ND	6.8c±0.3
10	André and Blaufränkisch	2.9a±0.2	3.1a±0.2	ND	ND	ND	ND	6.7b±0.3
11	Pinot noir	ND	ND	ND	ND	ND	ND	3.9a±0.2
12	Portugieser. Blauer	ND	1.1a±0.1	ND	ND	ND	ND	3.8b±0.2
13	Portugieser. Blauer	1.8a±0.1	3.4b±0.2	ND	ND	ND	ND	2.5c±0.1
14	Saint Laurent	ND	0.9a±0.1	ND	ND	ND	ND	6.2b±0.3
15	Zweigelt	ND	ND	ND	ND	ND	ND	4.4a±0.2
16	Zweigelt	ND	2.0a±0.1	ND	ND	ND	ND	4.9b±0.3
17	Zweigelt	ND	9.3a±0.4	ND	ND	ND	ND	7.8b±0.4

* ND – not detected
** HIS: histamine; TYR: tyramine; PUT: putrescine; CAD: cadaverine; PHE: phenylethylamine; SPD: spermidine; SPM: spermine
[a] The values are expressed as mean ± standard deviation. The mean values within a line (the difference between individual BA) followed by different letters differ ($p < 0.05$).

Table S3. Biogenic amines content (mg/L)[a] in red wines from Central European region.

Number	Wine	Biogenic amines content**						
		**HIS**	**TYM**	**PUT**	**CAD**	**PEA**	**SPD**	**SPN**
1	Alibernet	$1.2^{a}\pm0.1$	$3.9^{b}\pm0.2$	$5.9^{c}\pm0.3$	ND*	ND	ND	$3.9^{b}\pm0.2$
2	André	ND	$1.7^{a}\pm0.1$	$6.0^{b}\pm0.3$	ND	ND	ND	$3.9^{c}\pm0.2$
3	André	ND	$4.8^{a}\pm0.2$	ND	0.7b±0.1	$1.4^{c}\pm0.1$	$1.3^{c}\pm0.1$	$5.6^{d}\pm0.3$
4	André	ND	$1.8^{a}\pm0.2$	ND	ND	ND	ND	$6.6^{b}\pm0.3$
5	André	ND	$5.3^{a}\pm0.3$	ND	ND	ND	ND	$11.0^{b}\pm0.5$
6	Blaufränkisch	ND	$3.9^{a}\pm0.2$	$13.0^{b}\pm0.7$	1.5c±0.1	$1.5^{c}\pm0.1$	$1.1^{d}\pm0.1$	$8.3^{e}\pm0.4$
7	Blaufränkisch	$1.4^{a}\pm0.1$	$5.1^{b}\pm0.2$	$14.3^{c}\pm0.7$	0.8d±0.1	$1.9^{e}\pm0.1$	$0.6^{d}\pm0.1$	$3.2^{f}\pm0.1$
8	Blaufränkisch	ND	ND	ND	ND	ND	$1.2^{a}\pm0.1$	$5.6^{b}\pm0.3$
9	Blaufränkisch	$3.5^{a}\pm0.2$	$11.0^{b}\pm0.6$	$17.1^{c}\pm1.0$	ND	ND	ND	$6.1^{d}\pm0.3$
10	Blaufränkisch	$3.0^{a}\pm0.1$	$1.7^{b}\pm0.1$	$39.2_{c}\pm2.1$	ND	ND	ND	$3.0^{e}\pm0.1$
11	Cabernet Sauvignon	ND	$4.5^{a}\pm0.2$	$6.7^{b}\pm0.3$	ND	ND	ND	$9.2^{c}\pm0.4$
12	Cabernet Sauvignon	$9.6^{a}\pm0.4$	$2.9^{b}\pm0.2$	$6.5^{c}\pm0.3$	ND	$2.3^{d}\pm0.1$	$0.7^{e}\pm0.1$	$3.1^{b}\pm0.2$
13	Cabernet Sauvignon	ND	ND	ND	ND	ND	ND	ND
14	Dornfelder	ND	$4.9^{a}\pm0.2$	ND	ND	ND	ND	$5.5^{b}\pm0.3$
15	Dornfelder	ND	$4.0^{a}\pm0.2$	$8.9^{b}\pm0.4$	3.9a±0.2	ND	$3.9^{a}\pm0.2$	$9.1^{b}\pm0.4$
16	Merlot	ND	$6.1^{a}\pm0.3$	$11.1^{b}\pm0.6$	ND	ND	ND	ND
17	Merlot	$19.4^{a}\pm1.0$	ND	$272.0^{b}\pm12.8$	ND	$17.8^{c}\pm1.1$	$5.7^{d}\pm0.3$	ND
18	Merlot	$2.7^{a}\pm0.1$	$5.2^{b}\pm0.2$	$13.9^{c}\pm0.6$	ND	ND	ND	$6.8^{d}\pm0.4$
19	Mixture of Alibernet and Rubinet	$11.5^{a}\pm0.6$	$8.5^{b}\pm0.5$	$21.4^{c}\pm1.1$	ND	ND	ND	$3.0^{d}\pm0.2$
20	Mixture of Blaufränkisch and Saint Laurent and Portugieser. Blauer	$5.6^{a}\pm0.3$	$9.0^{b}\pm0.5$	$26.6^{c}\pm1.2$	ND	ND	ND	ND
21	Mixture of Cabernet Moravia and Alibernet	$3.0^{a}\pm0.2$	$6.2^{b}\pm0.3$	$12.0^{c}\pm0.6$	ND	$1.5^{d}\pm0.1$	ND	$5.5^{e}\pm0.3$
22	Pinot noir	ND	$18.1^{a}\pm0.9$	$6.6^{b}\pm0.3$	ND	ND	$1.3^{c}\pm0.1$	$6.6^{b}\pm0.3$
23	Pinot noir	ND	$1.3^{a}\pm0.1$	ND	ND	ND	ND	$5.6^{b}\pm0.3$
24	Portugieser. Blauer	ND	$4.9^{a}\pm0.2$	$7.3^{b}\pm0.4$	$1.4^{c}\pm0.1$	ND	$1.6^{c}\pm0.1$	$8.1^{d}\pm0.4$

25	Portugieser. Blauer	ND	ND	ND	ND	ND	ND	4.8^{a}±0.3
26	Portugieser. Blauer	ND	1.5^{a}±0.1	5.1^{b}±0.2	1.2^{c}±0.1	ND	ND	4.7^{b}±0.2
27	Portugieser. Blauer	ND	ND	17.6^{a}±0.9	ND	ND	ND	6.3^{b}±0.3
28	Portugieser. Blauer	ND	3.9^{a}±0.2	ND	ND	ND	ND	10.7^{b}±0.5
29	Portugieser. Blauer	15.3^{a}±0.8	16.2^{a}±0.8	74.0^{b}±3.8	ND	ND	ND	ND
30	Saint Laurent	ND	3.7^{a}±0.2	7.1^{b}±0.4	ND	ND	ND	6.8^{b}±0.3
31	Saint Laurent	ND	ND	ND	ND	ND	ND	3.9^{a}±0.2
32	Saint Laurent	1.8^{a}±0.1	2.0^{a}±0.1	8.4^{b}±0.4	ND	ND	6.8^{c}±0.3	3.5^{d}±0.2
33	Saint Laurent	2.3^{a}±0.1	25.9^{b}±1.3	8.0^{c}±0.4	ND	ND	ND	5.7^{d}±0.3
34	Saint Laurent	ND	ND	ND	ND	ND	ND	6.7^{a}±0.3
35	Zweigelt	ND	1.8^{a}±0.1	5.1^{b}±0.2	ND	ND	ND	6.4^{c}±0.4

* ND – not detected

** HIS: histamine; TYR: tyramine; PUT: putrescine; CAD: cadaverine; PHE: phenylethylamine; SPD: spermidine; SPM: spermine

[a] The values are expressed as mean ± standard deviation. The mean values within a line (the difference between individual BA) followed by different letters differ ($p < 0.05$).
